# Hippocampal µ-opioid receptors on GABAergic neurons mediate stress-induced impairment of memory retrieval

**DOI:** 10.1038/s41380-019-0435-z

**Published:** 2019-05-29

**Authors:** Mei-Mei Shi, Ka-Min Fan, Yan-Ning Qiao, Jin-Hui Xu, Li-Juan Qiu, Xiao Li, Ying Liu, Zhao-Qiang Qian, Chun-Ling Wei, Jing Han, Juan Fan, Ying-Fang Tian, Wei Ren, Zhi-Qiang Liu

**Affiliations:** 1 MOE Key Laboratory of Modern Teaching Technology, Center for Teacher Professional Ability Development, Xi’an, China; 2 School of Psychology, Shaanxi Normal University, Xi’an, China; 3 College of Life Sciences, Shaanxi Normal University, No.199, South Chang’an Rd., Xi’an, 710062 China

**Keywords:** Neuroscience, Psychology

## Abstract

Stressful life events induce abnormalities in emotional and cognitive behaviour. The endogenous opioid system plays an essential role in stress adaptation and coping strategies. In particular, the µ-opioid receptor (μR), one of the major opioid receptors, strongly influences memory processing in that alterations in μR signalling are associated with various neuropsychiatric disorders. However, it remains unclear whether μR signalling contributes to memory impairments induced by acute stress. Here, we utilized pharmacological methods and cell-type-selective/non-cell-type-selective μR depletion approaches combined with behavioural tests, biochemical analyses, and in vitro electrophysiological recordings to investigate the role of hippocampal μR signalling in memory-retrieval impairment induced by acute elevated platform (EP) stress in mice. Biochemical and molecular analyses revealed that hippocampal μRs were significantly activated during acute stress. Blockage of hippocampal μRs, non-selective deletion of μRs or selective deletion of μRs on GABAergic neurons (μR_GABA_) reversed EP-stress-induced impairment of memory retrieval, with no effect on the elevation of serum corticosterone after stress. Electrophysiological results demonstrated that stress depressed hippocampal GABAergic synaptic transmission to CA1 pyramidal neurons, thereby leading to excitation/inhibition (E/I) imbalance in a μR_GABA_-dependent manner. Pharmaceutically enhancing hippocampal GABA_A_ receptor-mediated inhibitory currents in stressed mice restored their memory retrieval, whereas inhibiting those currents in the unstressed mice mimicked the stress-induced impairment of memory retrieval. Our findings reveal a novel pathway in which endogenous opioids recruited by acute stress predominantly activate μR_GABA_ to depress GABAergic inhibitory effects on CA1 pyramidal neurons, which subsequently alters the E/I balance in the hippocampus and results in impairment of memory retrieval.

## Introduction

Severe or prolonged stressful life events impair cognitive function by influencing a series of brain regions in a complex manner [1, 2]. The hippocampus, a structure implicated in learning and memory processes, is particularly vulnerable to stressful experiences. Exposure to acute stress consistently disrupts hippocampal-dependent memory retrieval immediately afterward [3, 4]. Extensive studies have demonstrated that elevated glucocorticoid hormones during stress impair memory retrieval by modulating glutamatergic synaptic plasticity at the Schaffer collateral/commissural-CA1 synapses in the hippocampus, likely through inhibiting LTP induction or facilitating LTD [4–8]. Nevertheless, the magnitude of impairment by glucocorticoids does not match the impact of stress on memory [9], indicating that multiple neural mechanisms, not limited to the effects of glucocorticoids, may underlie stress-induced impairment of memory retrieval.

In addition to glucocorticoid hormones, acute stress also elicits the engagement of various neurotransmitters and neuromodulators, including monoamines, endogenous opioid peptides (EOPs), endocannabinoids, neuropeptide Y, oxytocin, and sex hormones, which interact with glucocorticoid hormones to manipulate responses to stress [9, 10]. Exposure to stress rapidly increases the release of enkephalin, β-endorphin, dynorphin, and nociceptin in brain regions closely related to emotion and cognition [11, 12]. Through activation of the three major opioid receptors that are densely expressed in the central nervous system (μ, δ, and κ), EOPs actively participate in the modulation of a series of cognitive, emotional, cardiovascular, and gastroenterological responses and enable adaptation to stress [13, 14]. However, whether the endogenous opioid system (EOS) plays an essential role in stress-induced impairments of learning and memory remains completely unknown.

Area CA1, a subfield of the hippocampus that receives substantial input from layer III of the entorhinal cortex via direct temporoammonic projections and Schaffer collaterals input from CA3, is necessary for the rapid formation and retrieval of spatial memory [15]. Opioid receptors are abundantly expressed on interneurons in CA1 [16]. Exogenously activating the CA1 μ-opioid receptors (μRs) reduces the firing rate and neurotransmitter release rate of the interneurons, which then disinhibit the pyramidal neurons, resulting in excitation/inhibition (E/I) imbalance [17–20]. Bias from the E/I balance could further disrupt the input-output functions of hippocampal synaptic circuits [21] and might impair spatial learning and memory. We thus hypothesize that, in addition to the direct effects of glucocorticoids on hippocampal glutamatergic synaptic plasticity, increased EOPs may also regulate stress-induced impairment of memory retrieval by inhibiting GABAergic interneurons in CA1. Such a regulatory mechanism of EOPs in local inhibitory circuitry in the hippocampus would be a novel pathway underpinning stress-induced memory impairments.

## Materials and methods

### Animals

Male C57BL/6J (from the Model Animal Research Center of Nanjing University, China) and mutant mice (8–12 weeks old) weighing 18–24 g were used in experiments. All experiments were carried out in accordance with the requirements of the Chinese Council on Animal Care and approved by the Animal Care Committee of Shaanxi Normal University. Mice were housed in groups of 4 in individually ventilated cages and maintained at 22 ± 2 °C and 55 ± 5% relative humidity under a 12:12-h light/dark schedule. Food and water were available ad libitum. The animals were handled for 5 days (3 min each day) before the behavioural experiments. All behavioural experiments and blood or tissue sample collection were conducted between 10:00 am and 3:00 pm. Animals were randomly assigned to groups without pre-estimating sample size.

*Oprm1-*floxed mice were constructed by inserting two loxP sites flanking exons 2–3 of the *Oprm1* gene. The *Oprm1* gene targeting vector was constructed with BAC retrieval methods. The *Oprm1* gene locus was retrieved from BAC clones obtained from a mouse C57BL/6 genomic library using a retrieval vector containing 2 homologous arms. The targeting vector included the 5′ region, a loxP site, the coding sequence of exons 2–3, a neomycin phosphotransferase expression (Neo) cassette (Frt-Neo-Frt), a second loxP site, and the 3′ region. After digestion by AscI, the linearized vectors were electroporated into C57BL/6 embryonic stem (ES) cells, and the targeted clones were selected with Southern blot analysis. Once homologous recombination was completed, the Sacl-digested-fragment and the EcoRI-digested-fragment would turn from 16.3 to 10.4 kb and from 15.1 to 10.2 kb, respectively, as detected with probes. Random integrations were negatively selected with diphtheria toxin A (DTA), and the positive clones were screened with G418. Selected ES clones were injected into blastocysts of C57BL/6 mice to generate chimeric mice. Germ-line transmission was obtained when chimaeras were crossed to B6 breeders. The Frt-Neo-Frt cassette was deleted by crossing with a Flp recombinase mouse. Floxed and wild-type *Oprm1* alleles in the offspring were detected by PCR using the following primers: *Oprm1*-A1 LoxP-F: 5′-GGTTAGAGTTAGGAGAATCAGGAGTTCAAG-3′; *Oprm1*-A2 LoxP-R: 5′-GTGAGAGTTGATGTTTGTAATTGAGTGCC-3′, product 214 bp (wild type) and 268 bp (floxed); *Oprm1*-Frt-F 5′-TAGTGTTAGGAAGATGTGCCATAG-3′ and *Oprm1*-Frt-R 5′-GATCAGAGTAACTGTCTTGGCTAC-3′, product 198 bp (wild type) and 328 bp (floxed). The *Oprm1*-floxed mice were then crossed to CMV-Cre mice [22] (from the Model Animal Research Center of Nanjing University, China) or mice expressing Cre in a cell-type-specific manner (including GFAP-CreERT2 mice [23], vGlut1-iCreERT2 mice and Gad2-iCreERT2 mice [24] from Beijing Biocytogen, China) as described previously in order to target *Oprm1* in all tissues, astroglial cells (µR_Astro_), glutamatergic neurons (µR_Glut_) or GABAergic neurons (µR_GABA_), respectively. The *Oprm1*^*loxP/loxP*^*::Gad2-iCreERT2*, *Oprm1*^*loxP/loxP*^*::vGlut1-iCreERT2*, or *Oprm1*^*loxP/loxP*^*::GFAP-CreERT2* mice and their control littermates (*Oprm1-flox+/+::CreERT2-/-*) were treated with tamoxifen (Sigma; dissolved in corn oil; 2 mg/day, i.p.) for 7 consecutive days to induce µR-specific deletion. Two weeks after the last tamoxifen injection, the mice were used for experiments. The deletion of µRs in mutant mice was verified by an *in situ* hybridization assay.

### Surgery and intra-hippocampal injection

Mice were anaesthetized with 0.8–1.5% isoflurane from a vaporizer (Fluotec-Ohmeda, Tewksbury, MA, USA) and fixed on a stereotaxic apparatus (SR-5; Narishige, Tokyo, Japan). Guide cannulas (23 gauge with stylets) made of stainless steel tubing were implanted into the bilateral dorsal hippocampus (anteroposterior, −1.8 mm from bregma; mediolateral, ±1.5 mm from bregma; dorsoventral, −1.0 mm from brain surface). Mice were allowed 7 days of recovery before the behavioural experiments. Intra-hippocampal injection was performed by insertion of needles (30 gauge) through the cannulas. The injection needles were connected to 1-µl syringes with polyethylene tubes and controlled by an automated microinjection pump (World Precision Instruments, Sarasota, FL, USA). Drug solutions were administered in a total volume of 1.0 µl/mouse (0.5 µl on each side) at a rate of 0.1 µl/min. After injection, the needles were left in place for an additional 2 min to allow drug diffusion. At the end of the experiment, mice were sacrificed with a urethane overdose, and the brains were sliced to verify the cannula placements. The data were abandoned if the cannula tip was >0.5 mm away from the correct placement. Naloxone, DAMGO, CTAP, naltrindole and nor-binaltorphimine were purchased from Tocris Bioscience, and bicuculline, L-655,708, and muscimol were purchased from Sigma. β-Endorphin antiserum (#ab10339), enkephalin antiserum (#ab77273) and control serum were provided by Abcam. All chemicals were dissolved in physiological saline unless otherwise stated.

### Elevated platform (EP) stress protocol

Stress was applied to mice by placing them on an elevated circular Plexiglas platform (1.3 m high, 8 cm diameter) in a brightly lit room for 50 min, whereas the control mice stayed in their home cages in the same room. Mice consistently urinated and/or defecated while on the platform. Immediately after stress was applied, mice were placed in a water maze for a memory-retrieval test or rapidly decapitated for either electrophysiological recording or collection of blood and brain tissue for corticosterone and Western blot analysis, respectively.

### Behavioural tests

#### Morris water maze (MWM) test

The apparatus was a circular pool (40 cm high and 100 cm diameter) filled with 21 ± 1 °C water (depth of 20 cm) that was made opaque with non-toxic white tempera paint powder. Visual landmarks with different colours and dimensions were placed near the pool for spatial orientation. The maze was divided into four imaginary quadrants. For hidden-platform training (on Day 1), a circular platform (10 cm diameter) was hidden 1 cm below the water surface (invisible to the mice) in the centre of one quadrant (the target quadrant) and remained in that location throughout training. Mice received a single training session consisting of twelve consecutive trials with four different starting positions that were evenly distributed around the perimeter of the maze. If a mouse reached the platform within 60 s, it was allowed to remain there for 20 s and was then placed in a holding cage for 5 min until the next trial. If a mouse searched for the platform for >60 s, it was guided to the platform gently by the experimenter. After completion of training, the animals returned to their home cages until probe testing 24 h later. The probe trial consisted of a 60-s free-swim period with the platform removed. The mice were placed in the pool at a position equidistant from the target quadrant and the opposite quadrant. Swimming paths for all trials were monitored using a video camera system. The escape latency (latency to find the platform) during the training section and the time spent in the four different quadrants during the test trial were analysed using an EthoVision tracking system (Noldus, Leesburg, VA, USA). To compare memory retrieval among different groups, we calculated the ratio of the time spent in the target quadrant to the value that would be expected by chance (15 s).

#### Open-field (OF) test

To assess the possible effects of µR deletion on motor and emotional activities, we evaluated locomotor activity and emotional response in the OF test. Mice were gently placed individually in a corner of the OF box (40 × 40 × 30 cm), facing the opaque walls, and allowed to freely explore the box for 10 min. The total distance travelled and the time spent in the centre area (20 × 20 cm) were automatically recorded using a video camera system and analysed with EthoVision software (Version 1.9, Noldus Information Technology, USA).

### ELISA assays of Met/Leu-enkephalin and corticosterone

Hippocampal Met/Leu-enkephalin concentrations and serum corticosterone levels were determined using the Mouse Enkephalin ELISA Kit (#abx576248, Abbexa, Ltd., Cambridge Science Park, UK) and Corticosterone ELISA Kit (#ADI-901–097, Enzo Life Sciences, Farmingdale, New York, USA) according to the manufacturer’s instructions.

### Electrophysiological recordings

Brain slices were prepared from mice that were anaesthetized with isoflurane and rapidly decapitated. The brains were chilled in oxygenated 4 °C modified artificial cerebrospinal fluid (ACSF), which contained the following (in mM): 250 sucrose, 2.5 KCl, 1.2 MgCl_2_, 1.2 NaH_2_PO_4_, 2.4 CaCl_2_, 26 NaHCO_3_ and 11 glucose. Transverse slices (300 μm) were cut with a vibratome (1000 plus; Vibratome Company, St. Louis, MO, USA). Slices were incubated in oxygenated standard ACSF for 1 h at room temperature (23–25 °C) to recover and then transferred to a recording chamber. The standard ACSF contained the following (in mM): 126 NaCl, 2.5 KCl, 1.25 NaH_2_PO_4_, 2 MgCl_2_, 2 CaCl_2_, 10 glucose and 26 NaHCO_3_, pH 7.4. CA1 pyramidal neurons were visualized with an upright microscope (DM LFSA, Leica, Germany). Whole-cell recordings were performed using a Multiclamp 700B amplifier (Molecular Devices, Sunnyvale, CA, USA). The recording pipettes had 3–5 MΩ resistance when filled with the following solution (in mM): 80 CsCH_3_SO_3_, 80 CsCl, 10 HEPES, 2 QX-314, 2 MgCl_2_, 0.2 EGTA, 4 MgATP, 0.3 Na_2_GTP, 10 Na_2_-phosphocreatine (pH 7.2 with CsOH).

Evoked excitatory postsynaptic currents (eEPSCs) and inhibitory postsynaptic currents (eIPSCs) were induced with a concentric bipolar electrode placed in the Schaffer collateral pathway, which was stimulated at 0.1 Hz (pulse of 100 μs duration). Input/output (I/O) curves were constructed by varying the stimulus intensity (10–500 mA) from a stimulation isolation unit controlled by a S88X stimulator (Grass Technologies, West Warwick, RI, USA) and measuring the peak eEPSC/eIPSC amplitude. eEPSCs were recorded in the presence of the GABA_A_ receptor antagonist picrotoxin (PTX, 100 μM, Sigma). eIPSCs were recorded in the presence of the glutamate receptor antagonists D-AP5 (50 μM, Sigma) and DNQX (20 μM, Sigma). The eEPSC and eIPSC responses were verified by bath application of PTX (100 μM) and DNQX (20 μM), respectively. Individual data were normalized to the respective maximal response. For analysis of paired-pulse facilitation/depression of eIPSC amplitude, the neuron was voltage clamped at −70 mV, and pairs of stimuli were applied at varying interpulse intervals (50, 100, and 200 ms). The paired-pulse ratio (PPR) was calculated by determining the ratio of the peak amplitude values of evoked eIPSC2/eIPSC1 by paired-pulse stimulation at each interpulse interval. To obtain eEPSC/eIPSC (E/I) ratios under standard ACSF bath conditions, we used the average of the maximal peak from 10 eEPSCs (voltage clamped at −70 mV) and the average of the maximal peak of 10 eIPSCs (voltage clamped at 0 mV). Spontaneous inhibitory postsynaptic currents (sIPSCs) and miniature inhibitory postsynaptic currents (mIPSCs) were recorded at a holding potential of −70 mV with 50 μM D-AP5 and 20 μM DNQX in standard ACSF. For mIPSC recordings, 1 µM tetrodotoxin (Fishery Science and Technology Development Co., China) was added. sIPSCs and mIPSCs were analysed starting 4–10 min from the beginning of whole-cell recording. Before the tonic inhibitory current was measured, the baseline current was stabilized with 50 μM D-AP5 and 20 μM DNQX to block AMPA and NMDA-receptor-dependent postsynaptic currents. The amplitude of the tonic GABA current was measured by the baseline shift after 100 μM bicuculline administration. Tonic current density was calculated from the current amplitude divided by the membrane capacitance.

### Western blotting analysis

Hippocampal slice surface proteins were labelled with EZ-Link Sulfo-NHS-SS-Biotin as described previously [25]. Briefly, after recovery, slices were incubated with ACSF containing 1 mg/ml EZ-Link Sulfo-NHS-SS-Biotin (#21331, Pierce Chemical Co., Rockford, IL, USA) for 45 min. Then, the slices were rinsed 3 times with oxygenated ACSF containing glycine, homogenized in 500 μl of modified radioimmunoprecipitation assay buffer (50 mM Tris-HCl, pH 8; 150 mM NaCl; 1% Triton X-100 and 1% sodium deoxycholate; 10 μg/ml leupeptin; 100 μg/ml TPCK; and 1 mM PMSF), and then centrifuged at 14,000×*g* for 15 min. Proteins (150–300 μg) were incubated overnight in an end-over-end shaker in the presence of Streptavidin beads (#20349, Pierce Chemical Co., Rockford, IL, USA). The entire process was carried out at 4 °C. The Beads were thoroughly washed, resuspended in 30 μl loading buffer, and analysed by Western blots as described below.

Protein concentrations were determined by the Lowry method. Protein samples were separated by 10% SDS-PAGE and transferred to a nitrocellulose membrane. After being blocked at room temperature in 10% milk in TBST buffer (10 mM Tris-HCl, 120 mM NaCl, and 0.1% TWEEN 20, pH 7.4) for 1 h, the membrane was probed with antibodies against μR (1:500; #ab10275, Abcam, USA), phospho-μR (Ser375, 1:1 000; #bs-3724R, BIOSS, USA) and β-actin (1:1 000; #4970, Cell Signalling Technology, USA) at 4 °C overnight. The membranes were then washed three times in TBST, followed by incubation with 1:10,000 dilutions of horseradish peroxidase-conjugated anti-rabbit/mouse IgG at room temperature for 1 h and washed three times in TBST. Visualization was carried out using a chemiluminescence kit (Bio-Rad). The density of the bands was quantified by densitometric analysis of the scanned blots using Bio-Rad software. Rat μR peptide (#ab46988, Abcam, USA) was used to verify the specificity of the μR antibody (#ab10275, Abcam, USA) by Western blot. After being blocked with 10% milk in TBST, the membrane was incubated with primary antibody or mixture (antibody mixed with peptide; antibody:peptide = 1:2) overnight at 4 °C.

### Fluorescence in situ hybridization with RNAscope

The deletion of µRs in mutant mice was verified by fluorescent in situ hybridization assay. Briefly, mice were deeply anaesthetized with isoflurane and sacrificed by perfusion with 4 °C saline (0.9%, pH 7) within 5 min. The brains were quickly removed from the skulls and frozen on dry ice and then embedded in OCT (#Tissue-Tek 4583, Sakura Finetek USA Inc., Torrance, CA). Fresh frozen sections (16 μm) were made coronally through the hippocampal formation with a freezing microtome (CM1950, Leica Microsciences, Germany) and thaw mounted onto Superfrost Plus Microscope Slides (#12–550–15, Fisher Scientific, Pittsburgh, PA, USA). The sections were fixed in 4% PFA for 60 min at 4 °C before being dehydrated using graded ethanol (50, 70, 100 and 100%) at room temperature for 5 min each and finally air dried. The sections were incubated with H_2_O_2_ for 10 min and subsequently pretreated with protease IV for 15 min. The probes for *Oprm1* (sixteen synthetic oligonucleotides complementary to the nucleotide sequence 590–1458 of *Oprm1*), Gad2 (#39371), vGlut1 (#416631) and GFAP (#313211) were provided by Advanced Cell Diagnostics (ACD, USA) and conjugated to Atto 550 and Atto 647, respectively. The procedure for in situ detection was performed using RNAscope Multiplex Fluorescent Reagent Kit v2 (#323100, ACD) according to the manufacturer’s instructions for fresh frozen tissue. After being heated with a HybEZTM oven (ACD, USA) for 2 h, slides were mounted with the ProLong Gold Antifade Mountant (#P10144, Thermo Fisher Scientific, Pittsburgh, PA, USA). Confocal images were captured with a laser scanning microscope (Leica, TCS SP5, Germany), and cells with positive labelling were counted.

### Statistical analysis

Data were presented as the mean ± standard error of the mean (SEM), or sometimes as the mean ± SEM %, and analysed (not in a blinded manner) using SPSS 20. Normality was tested with the Shapiro-Wilk test, and equal variance was evaluated before ANOVA. Statistical analysis of the data was performed using Student’s *t*-test (two-tailed), the Kolmogorov-Smirnov (K-S) test, one-way analysis of variance (ANOVA) or repeated-measures (RM) ANOVA, as stated individually in the results section. The ANOVAs were followed by post hoc tests (Fisher’s test). Statistical significance was set at *p* < 0.05.

## Results

During the 12 trials of training in the MWM task, the escape latencies of all naïve mice (*n* = 41) progressively decreased, showing successful spatial memory acquisition (Supplementary Figure 1a). Twenty-four hours later, the trained mice were randomly divided into the unstressed (*n* = 21) and stressed groups (*n* = 20). Both groups were subjected to the probe test, with the stressed group undergoing 50 min of EP stress immediately prior to the probe test. Although no difference in motor capacity, shown as average swimming speed, was observed between the two groups (Supplementary Figure 1b), the unstressed mice performed well and spent significantly longer in the target quadrant than in the opposite quadrant (23.33 ± 1.58 s vs. 7.01 ± 0.78 s), whereas the stressed mice spent similar amounts of time in each of those two quadrants, spending significantly less time in the target quadrant than the unstressed mice (Supplementary Figure 1c). These results demonstrate that acute EP stress before the probe test significantly impairs the retrieval of spatial reference memory. In addition, such impairment effects of EP stress on memory retrieval lasted for at least 2 h (Supplementary Figure 1d).

### Hippocampal µRs are necessary for stress-induced impairment of memory retrieval

**Fig. 1:**
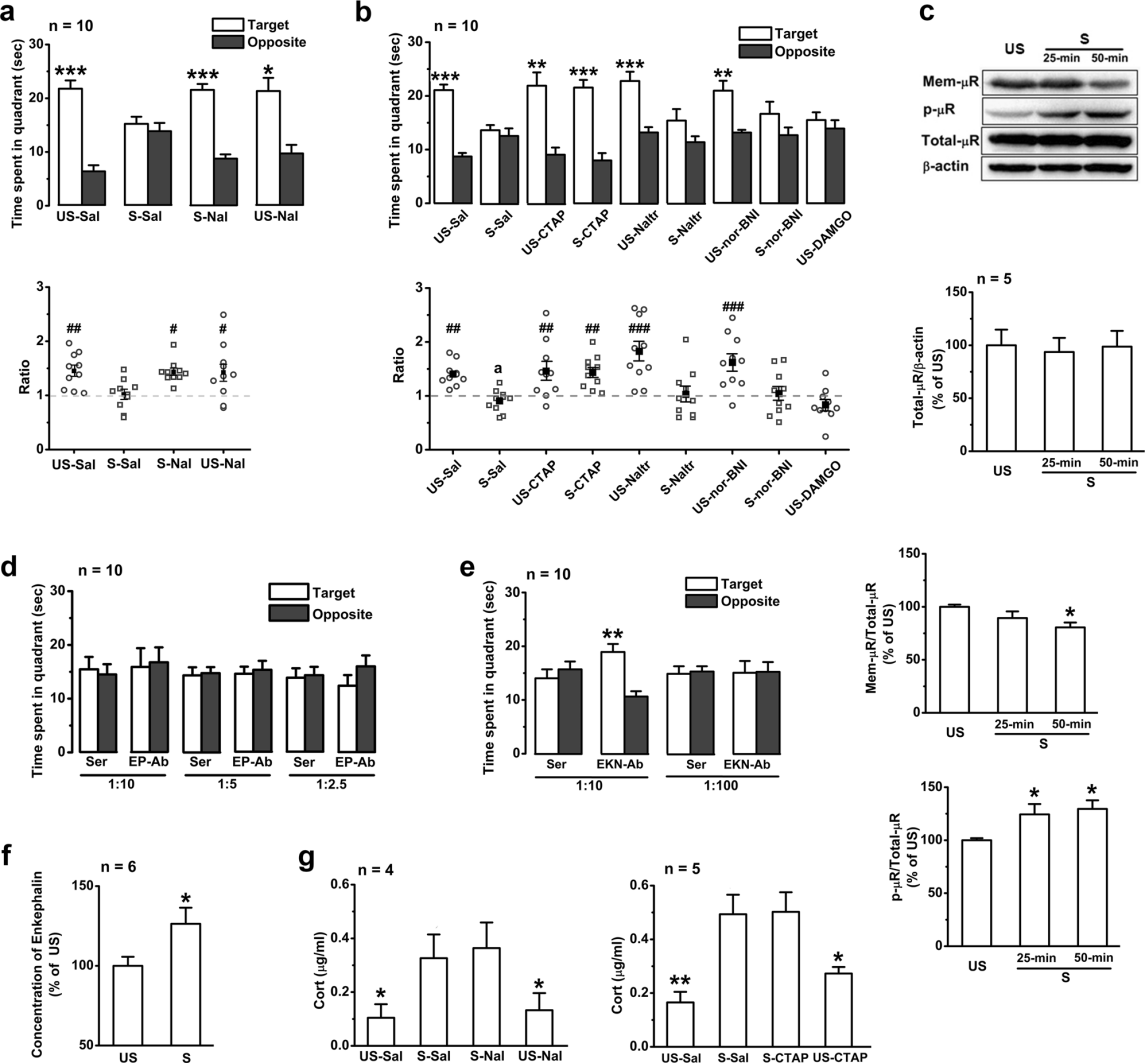
EP stress impairs memory retrieval in the MWM task by activating hippocampal µRs. **a** The effects of systemic injection of naloxone or saline on time spent in the target/opposite quadrants and the target time ratio during probe test of the stressed and unstressed mice. *, target vs. opposite within-group, paired Student’s *t*-test. The target time ratio *F*_3,36_ = 3.59, *p* *=* 0.023, one-way ANOVA; #, vs. S-Sal. **b** The effects of bilateral intra-hippocampal infusion of CTAP (0.5 µg/µl), naltrindole (2.26 µg/µl), nor-binaltorphimine (3.85 µg/µl), DAMGO (0.5 μg/μl), or saline on memory retrieval. The target time ratio *F*_8,81_ = 7.00, *p* *=* 0.000, one-way ANOVA; #, vs. S-Sal. **c** The effects of EP stress on the levels of hippocampal µR proteins and their phosphorylation. Total µRs *F*_2,15_ = 0.25, *p* *=* 0.784; cell surface µRs *F*_2,12_ = 4.09, *p* *=* 0.044; phosphorylated µRs *F*_2,12_ = 4.75, *p* *=* 0.030; one-way ANOVA; *, vs. unstressed. **d** Hippocampal injection of β-endorphin antiserum does not affect stress-induced memory impairment. **e** Hippocampal injection of enkephalin antiserum at a concentration of 1:10 before stress abolishes memory impairment. **f** The level of Met/Leu-enkephalin in hippocampal tissues. **g** The effects of systemic application of naloxone or bilateral intra-hippocampal infusion with CTAP on the level of serum corticosterone immediately after 50-min stress. *F*_3,12_ = 4.22, *p* *=* 0.030 for naloxone groups, and *F*_3,12_ = 8.74, *p* *=* 0.000 for CTAP groups, one-way ANOVA; *, vs. stress. One symbol, *p* < 0.05; two symbols, *p* < 0.01; three symbols, *p* < 0.001. *US* un-stress, *S* stress, *Sal* saline, *Nal* naloxone, *Naltr* naltrindole, *nor-BNI* nor-binaltorphimine, *Cort* corticosterone, *Mem-µR* cell surface µRs, *p-µR* phosphorylated µRs, *EKN-Ab* enkephalin antiserum, *EP-Ab* β-endorphin antiserum, *Ser* control serum

The nonspecific opioid receptor antagonist naloxone was first used (3 mg/kg, i.p.) to address the involvement of EOPs in the stress-induced impairment of memory retrieval. Intraperitoneal injection (i.p.) of naloxone (3 mg/kg) or its vehicle had no significant effect on the memory retrieval of unstressed mice (Fig. 1a), but naloxone injection 30 min before EP stress completely abolished the memory-retrieval impairment observed in the stressed mice (Fig. 1a), suggesting that the opioid receptors are required for this memory impairment. No significant difference was detected in swimming speed among the treated groups (Supplementary Figure 2a).

Three major opioid receptors, the µRs, δ-opioid receptors (δRs), and κ-opioid receptors (κRs), are expressed in the hippocampus. In order to specify the subtype of opioid receptors involved in the responses to EP stress, selective opioid receptor antagonists were then bilaterally microinjected into the hippocampal CA1 region 15 min before EP stress. Intra-hippocampal infusion of the µR antagonist CTAP, the δR antagonist naltrindole or the κR antagonist nor-binaltorphimine 65 min before the probe test did not affect the memory retrieval (Fig. 1b) of the unstressed mice (*p* > 0.05, compared with the saline group of unstressed mice). The application of CTAP (0.5 µg/µl), but not any dose of naltrindole (2.26 µg/µl or 4.52 µg/µl) or nor-binaltorphimine (3.85 µg/µl or 7.70 µg/µl), completely eliminated the stress-induced impairment of memory retrieval (Fig. 1b; Supplementary Figure 1e). The stressed mice in the CTAP group spent significantly more time in the target quadrant than the stressed mice in the saline, naltrindole, or nor-binaltorphimine group (21.54 ± 1.42 s vs. 13.59 ± 0.97, 15.40 ± 2.15, 16.65 ± 2.25 s, respectively). Furthermore, microinjection of DAMGO (0.5 μg/μl), a selective µR agonist, in the CA1 regions of the unstressed mice prior to the probe test reproduced memory-retrieval impairment similar to that observed in the stressed mice (Fig. 1b). Given that the acquisition was not affected by cannula implantation (Supplementary Figure 1a) and that swimming speed in the probe test was not affected by the administration of any of the drugs (Supplementary Figure 2b), it is reasonable to conclude that the EP-stress-induced impairment of memory retrieval specifically requires activation of µRs in the hippocampus.

Upon EOP binding on µRs, phosphorylation of the serine at position 375 (Ser375) on the C-terminal tail of µRs occurs and further promotes receptor internalization and trafficking [26]. We probed the levels of μRs and phospho-μRs (at Ser375 site) with the specific antibodies (Supplementary Figure 3). Consistent with the previous results obtained using an agonist [26], the levels of Ser375 phosphorylation in the hippocampus increased significantly after 25 or 50-min EP stress (Fig. 1c). Although the total amount of μR protein remained unchanged, the level of cell-surface µRs was significantly reduced 50 min but not 25 min after EP stress (Fig. 1c), suggesting that endocytosis of membrane µRs occurred following phosphorylation. These results consistently indicate the binding of EOPs with µRs in the hippocampus during EP stress.

The hippocampus is enriched with endorphinergic projections and enkephalinergic neurons that can release β-endorphin and Met/Leu-enkephalin, respectively, which have high binding affinity for µRs [27]. To test whether µR is endogenously activated by EOPs during stress and therefore impairs memory retrieval, we inhibited the function of β-endorphin and Met/Leu-enkephalin with their respective antiserums [28, 29]. Bilateral hippocampal microinjection with the β-endorphin antiserum at different dilutions (1:10, 1:5 and 1:2.5) 65 min before EP stress did not affect the impairment of memory retrieval (Fig. 1d), whereas microinjection of the Met/Leu-enkephalin antiserum at a concentration of 1:10 completely abolished the memory-retrieval impairment induced by EP stress (Fig. 1e). Furthermore, a significant increase in Met/Leu-enkephalin in hippocampal tissues was detected immediately after the stress (Fig. 1f). Microinjection of Met/Leu-enkephalin antiserum at 1:100 or its control serum did not show such an abolishing effect (Fig. 1e). Swimming speed in all treated groups (Supplementary Figures 4a-b) and memory retrieval in the unstressed groups (Supplementary Figure 4c) were not affected by antiserum or control serum injection. These results demonstrate that activation of µRs by endogenous enkephalin plays an important role in the memory-retrieval impairment induced by EP stress.

Given the critical role of corticosteroid hormones in stress-induced memory impairments [3, 7], it is possible that the µR inhibitors might reverse memory-retrieval impairment via inhibiting corticosterone release on CA1 glutamatergic synapses. To our surprise, neither systemic application of naloxone nor intra-hippocampal infusion of CTAP (Fig. 1g) affected the elevation of corticosterone levels caused by EP stress.

### Stress-induced impairment of memory retrieval depends on activation of μRs on GABAergic neurons

**Fig. 2:**
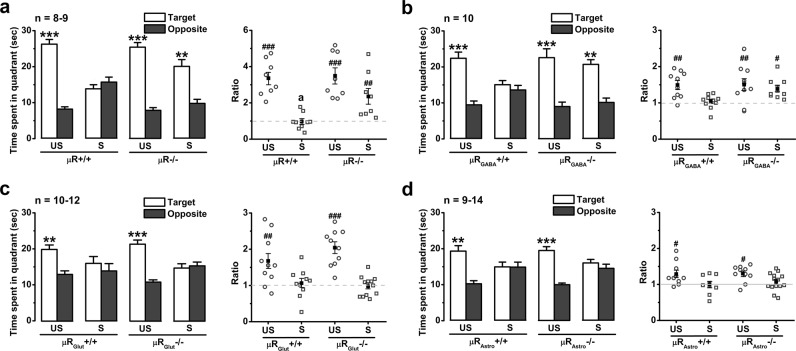
µR_GABA_ but not µR_Glut_ or µR_Astro_ deletion prevents the EP stress-induced impairment of spatial memory retrieval. **a**-**d** The effects of stress on memory retrieval of µR−/− (**a**), µR_GABA_−/− (**b**), µR_Glut_−/− (**c**), and µR_Astro_−/− mice (**d**). *, target vs. opposite within-group, paired Student’s *t*-test. The target time ratio *F*_3,29_ = 10.95, *p* *=* 0.000 for µR−/−; *F*_3,36_ = 3.76, *p* *=* 0.019 for µR_GABA_−/− mice; *F*_3,38_ = 11.86, *p* *=* 0.000 for µR_Glut_−/−; and *F*_3,39_ = 3.44, *p* *=* 0.026 for µR_Astro_−/−; one-way ANOVA. #, vs. the stressed µR+/+, µR_GABA_+/+, µR_Glut_+/+, or µR_Glut_+/+, respectively. One symbol, *p* < 0.05; two symbols, *p* < 0.01; three symbols, *p* < 0.001. *US* unstressed, *S* stressed

In the central nervous system, μRs are widely expressed in different cell types, including GABAergic inhibitory interneurons [16], astrocytes [30], and even glutamatergic neurons [31]. To further determine the functional site of EOPs in stress-induced impairment of memory retrieval, *Oprm1* flox (Supplementary Figure 5) mice were constructed to conditionally delete the *Oprm1* gene in different cell types. We developed four mouse lines lacking µRs in all tissues (referred to as µR−/−), GABAergic inhibitory interneurons (µR_GABA_−/−), glutamatergic neurons (µR_Glut_−/−), and astroglial cells (µR_Astro_−/−) (see ‘Methods and materials'). The fluorescent in situ hybridization studies confirmed the absence of µRs non-selectively in all tissues (Supplementary Figure 6) or selectively in GAD2-positive neurons, vGLUT1-positive neurons, and GFAP-positive cells (Supplementary Figure 7), respectively, in the CA1 region, suggesting the validity of µR deletions. In each of the knockout lines and their littermate control (µR+/+, µR_GABA_+/+, µR_Glut_+/+, and µR_Astro_+/+), no significant difference was observed in the memory acquisition (Supplementary Figures 8a-d) and retrieval of the MWM test (Figs. 2a-d), locomotion distance and time spent in the centre zone of the OF test (Supplementary Figures 8a-d), indicating that non-selective or cell-type-selective deletion of µRs does not affect learning and memory, emotion, or locomotor behaviour. In addition, no significant difference was found in swimming speed between any line of knockout mice and their littermate controls regardless whether they were stressed (Supplementary Figures 8a-d). Similar to the effects of naloxone systemic treatment (Fig. 1a), non-selective µR deletion prevented the stress-induced impairment of spatial memory retrieval (Fig. 2a). Furthermore, selective µR deletion from GABAergic neurons abolished the stress-induced impairment of memory retrieval, which remained in the µR_GABA_+/+ mice (Fig. 2b). On the other hand, selective µR deletion in glutamatergic neurons or in astroglial cells had no impact on the stress-induced memory impairment (Figs. 2c, d). Together with the results of hippocampal microinjection with µR antagonists and agonists, these results definitively reveal the essential role of µRs on GABAergic neurons in stress-induced memory impairment.

### EP stress depresses GABAergic synaptic transmission onto CA1 pyramidal neurons in a µR_GABA_-dependent manner

**Fig. 3:**
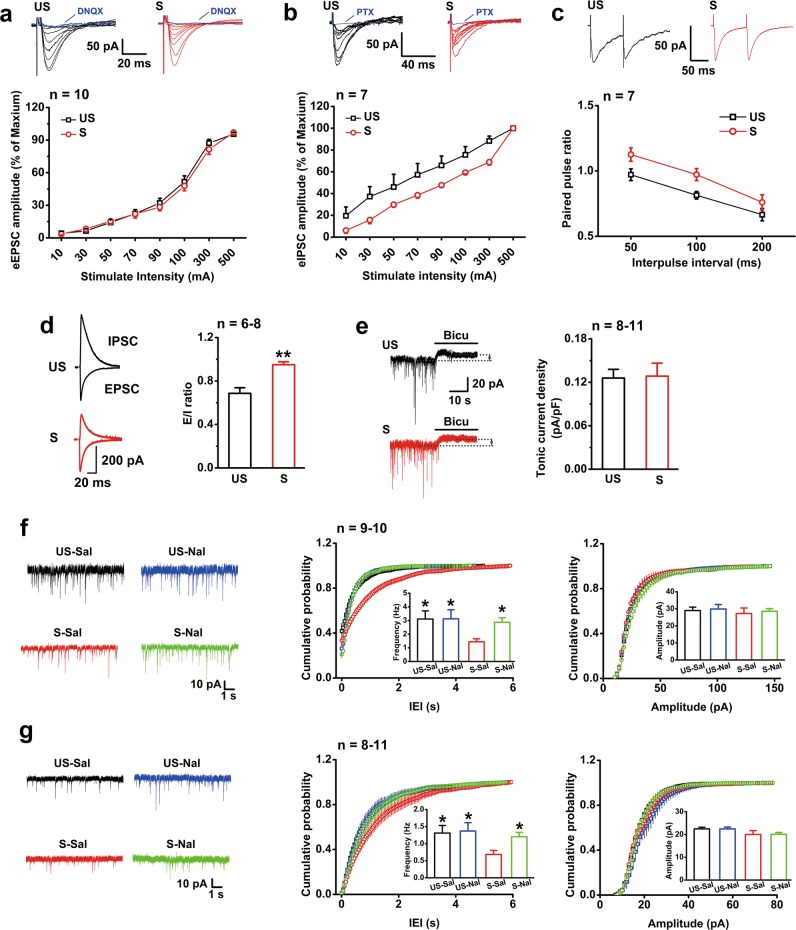
EP stress attenuates inhibitory synaptic transmission on CA1 pyramidal neurons. The number of cells labelled is equal to the number of animals used. **a** Stress does not alter the I/O curves of eEPSCs. The eEPSC was abolished by DNQX (20 μM). Group *F*_1,17_ = 0.558, *p* = 0.465; group×intensity *F*_9,153_ = 1.15, *p* = 0.332; RM ANOVA. **b** I/O curves of eIPSCs show a significant difference between the unstressed and stressed mice. The eIPSC was abolished by PTX (100 μM). Group *F*_1,12_ = 4.94, *p* = 0.046; group×intensity *F*_7,84_ = 1.55, *p* = 0.163; RM ANOVA. **c** PPR of eIPSCs at different interpulse intervals. Group *F*_1,12_ = 9.78, *p* = 0.009; group×interval *F*_2,24_ = 0.34, *p* = 0.717; RM ANOVA. **d** The E/I ratio is increased in the stressed mice. **e** The tonic inhibitory currents of CA1 individual pyramidal neurons in slices from the unstressed and stressed mice are assessed by application of bicuculline. Stress does not affect the density of tonic inhibitory current. **f** EP stress depresses sIPSCs in CA1 pyramidal neurons, and this effect is reversed by naloxone administration. *p* < 0.05 for cumulative probability of IEIs (S-Sal vs. US-Sal, US-Nal or S-Nal) and *p* > 0.05 for cumulative probability of amplitude, K-S test. Mean values of frequency *F*_3,35_ = 4.46, *p* *=* 0.021; and mean values of amplitude *F*_3,35_ = 0.19, *p* *=* 0.903; one-way ANOVA; *, vs. S-Sal. **g** EP stress depresses mIPSCs in CA1 pyramidal neurons, and this effect is reversed by naloxone administration. *p* < 0.05 for probability of IEIs (S-Sal vs. US-Sal, US-Nal or S-Nal) and *p* > 0.05 for probability of amplitude, K-S test. Frequency *F*_3,33_ = 2.93, *p* *=* 0.048; amplitude *F*_2,26_ = 1.72, *p* *=* 0.181; one-way ANOVA; *, vs. S-Sal. One symbol, *p* < 0.05; two symbols, *p* < 0.01. *US* unstressed, *S* stressed, *Bicu* bicuculline, *Sal* saline, *Nal* naloxone

The eEPSCs and eIPSCs of pyramidal neurons were recorded in vitro to investigate the changes in basal excitatory and inhibitory synaptic transmission, respectively, in CA1 slices that were prepared immediately after EP stress. The I/O curve of eIPSCs (Fig. 3b), but not of eEPSCs (Fig. 3a), shifted rightward significantly in the stressed mice compared with the unstressed mice. The PPR of eIPSCs in slices from the stressed mice was markedly larger than that from the unstressed mice (Fig. 3c), implying a presynaptic depression on the basal inhibitory transmission by EP stress. To evaluate the E/I ratio, we recorded eEPSCs (voltage clamped at −70 mV) and eIPSCs (voltage clamped at 0 mV) from identical CA1 pyramidal neurons. The results from the stressed mice exhibited a profound increase in the E/I ratio (Fig. 3d). These results suggest that EP stress leads to an E/I imbalance by reducing the inhibitory inputs to CA1 pyramidal neurons.

Unlike the tonic currents mediated by extrasynaptic GABA_A_ receptors, the phasic IPSCs evoked by spontaneous quantal release (including sIPSCs and mIPSCs) are mediated by intrasynaptic GABA_A_ receptors [32]. The tonic inhibitory currents, revealed by bath application of bicuculline (100 µM), showed no difference in CA1 pyramidal neurons between the stressed and unstressed mice (Fig. 3e). However, compared with the unstressed mice, the average frequency of sIPSCs in slices from the stressed mice was significantly depressed, and the cumulative distribution of their inter-event intervals (IEIs) was shifted rightward, while the average sIPSC amplitude remained unchanged (Fig. 3f). Similar changes were observed in the average frequency, average amplitude and cumulative distribution of IEIs in mIPSCs (Fig. 3g) of the stressed mice, together indicating that the neurotransmitter release from GABAergic interneurons onto pyramidal neurons was markedly depressed following EP stress.

**Fig. 4:**
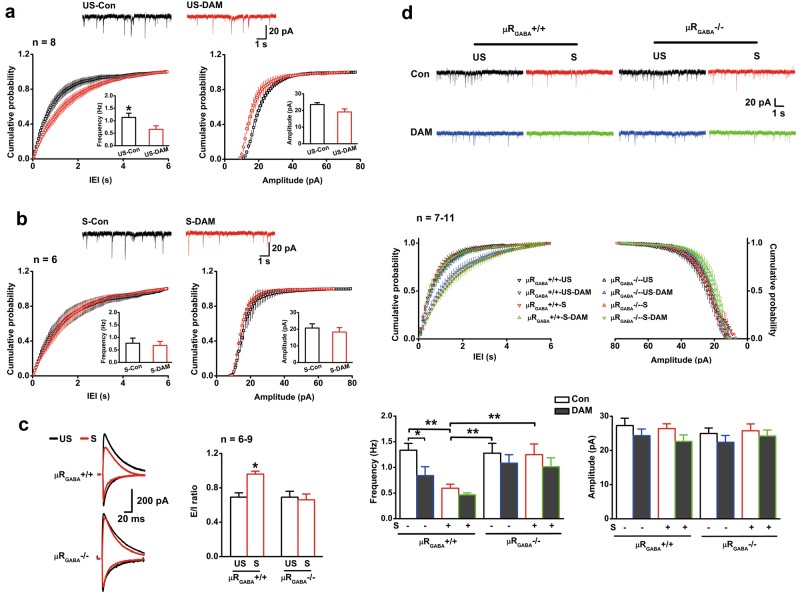
EP stress occludes DAMGO perfusion-induced depression of mIPSC frequency. The number of cells labelled is equal to the number of animals used. **a** DAMGO perfusion significantly decreases mIPSC frequency but does not change mIPSC amplitude of the unstressed mice. *p* < 0.05 for cumulative probability of IEIs, and *p* > 0.05 for cumulative probability of amplitude, K-S test. *p* *=* 0.019 for mean values of frequency and *p* = 0.098 for mean values of amplitude, paired Student’s *t*-test. **b** DAMGO perfusion does not influence the mIPSC frequency or amplitude of the stressed mice. *p* > 0.05 for probability of IEIs and amplitude, K-S test. *p* = 0.742 for mean values of frequency and *p* = 0.527 for amplitude, paired Student’s *t*-test. **c** The stress-enhanced E/I ratio is prevented by µR_GABA_ deletion. *F*_3,26_ = 4.73, *p* = 0.009; one-way ANOVA. *, vs. unstressed µR_GABA_+/+ or stressed µR_GABA_−/−. **d** Deletion of µR_GABA_ prevents the decrease in mIPSC frequency induced by EP stress or DAMGO perfusion. *p* < 0.05 for cumulative probability of IEIs (µR_GABA_+/+–US vs. µR_GABA_+/+–US–DAM, µR_GABA_+/+–S, or µR_GABA_+/+–S–DAM) and *p* > 0.05 for cumulative probability of amplitude, K-S test. mIPSC frequency *F*_7,64_ = 4.37, *p* *=* 0.000; mIPSC amplitude *F*_7,64_ = 0.85, *p* = 0.554; one-way ANOVA. One symbol, *p* < 0.05; two symbols, *p* < 0.01. *US* unstressed, *S* stressed, *Con* control before DAMGO perfusion, *DAM* DAMGO

We next asked whether this depression of GABAergic transmission results from activation of µRs during stress. Administration of naloxone (3 mg/kg, i.p.) did not alter the frequency and amplitude of sIPSCs (Fig. 3f) and mIPSCs (Fig. 3g) in the unstressed mice (*p* > 0.05, vs. saline mice). In contrast, naloxone injection prior to EP stress abolished the depression of the average frequencies of both sIPSCs (Fig. 3f) and mIPSCs (Fig. 3g), without significant influence on the average amplitudes. Moreover, bath application of specific µR agonist DAMGO (1 µM) in slices from the unstressed mice for 30 min was capable of reproducing a significant depression in mIPSC frequency (57.0% of baseline) similar to that observed in the stressed mice (55.8% of unstressed level) (Fig. 4a). Interestingly, bath application of DAMGO in the same way was no longer able to further depress mIPSC frequency in slices from the stressed mice (Fig. 4b), indicating that the inhibiting effects of µR activation on GABAergic transmission might have been saturated by endogenous EOPs released during EP stress. The depression of GABAergic transmission by activation of µRs was indicated to occur presynaptically at the GABAergic terminals because application of DAMGO had no detectable effect on the amplitude of mIPSCs in slices from both the unstressed and stressed mice (Figs. 4a, b). These data thus provide strong evidence that activation of µRs is required for the EP stress-induced depression of GABAergic inhibitory transmission on CA1 pyramidal neurons.

Given that the µRs on GABAergic neurons mediated stress-induced memory impairment (Fig. 2b), we then used µR_GABA_−/− mice to further verify the requirement of µR_GABA_ in the observed depression of hippocampal GABAergic transmission and E/I imbalance. No significant difference in the E/I ratio (Fig. 4c), mIPSC frequency or amplitude (Fig. 4d) was observed between the unstressed µR_GABA_−/− and µR_GABA_+/+ mice, showing that inducible µR_GABA_ deletion did not affect basal synaptic release or E/I balance. Bath application of DAMGO shifted the cumulative distribution of IEIs rightward and depressed mIPSC frequency only in slices from µR_GABA_+/+ mice and not slices from µR_GABA_−/− mice (Fig. 4d), indicating that the inhibitory function of µRs on GABAergic transmission is lacking in µR_GABA_−/− mice. As expected, stress-induced enhancement of the E/I ratio in µR_GABA_+/+ mice was no longer present in µR_GABA_−/− mice (Fig. 4c). Consistently, the depression of mIPSC frequency induced by EP stress observed in µR_GABA_+/+ mice was absent in µR_GABA_-/- mice (Fig. 4d).

These electrophysiological results convincingly revealed that hippocampal GABAergic transmission on pyramidal neurons is depressed as a consequence of the specific activation of µRs on GABAergic neurons during EP stress.

### Depression of GABAergic inhibitory transmission in the hippocampus impairs memory retrieval

**Fig. 5:**
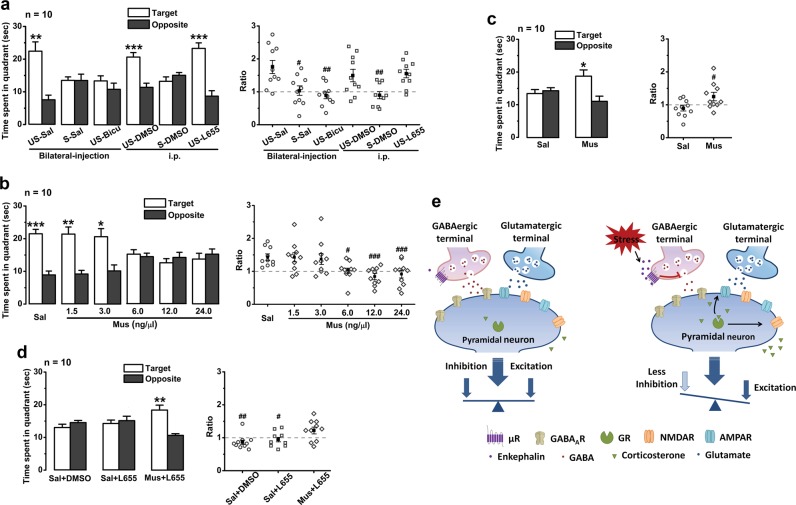
Intrasynaptic GABA_A_ receptor-mediated inhibitory currents modulate memory retrieval. Symbol *, target vs. opposite within-group, paired Student’s *t*-test. **a** Memory retrieval of unstressed mice is damaged by application of bicuculline but not L-655,708 or vehicle before probe test. The target time ratio *F*_5,54_ = 6.37, *p* = 0.001, one-way ANOVA; #, vs. US-vehicle (saline or DMSO). **b** The effects of hippocampal infusion of different doses of muscimol on memory retrieval in unstressed mice. The target time ratio *F*_5,54_ = 6.34, *p* = 0.000, one-way ANOVA; #, vs. Sal. (**c**) Muscimol (1.5 ng/1.0 µl) infusion immediately after stress rescues the impaired memory retrieval. **d** Blocking tonic inhibitory currents with L-655,708 does not disturb the rescuing effect of muscimol on stress-impaired memory retrieval. The target time ratio *F*_2,27_ = 5.28, *p* *=* 0.012, one-way ANOVA; #, vs. Mus + L655. One symbol, *p* < 0.05; two symbols, *p* < 0.01; three symbols, *p* < 0.001. *Sal* saline, *Bicu* bicuculline, *L655* L-655,708, *Mus* muscimol. **e** A schematic model for the two pathways in the hippocampus underlying the impairment of memory retrieval by acute stress. GR can directly modulate glutamatergic synaptic plasticity [4, 68] and affect memory retrieval. In addition, enkephalin triggered by acute stress in the hippocampus predominantly activates µR_GABA_ and then disinhibits pyramidal neurons, resulting in E/I imbalance and impairment of memory retrieval

The behavioural and electrophysiological results demonstrated that activation of µR_GABA_ depresses hippocampal GABAergic transmission on pyramidal neurons and therefore impairs spatial memory retrieval. If the stress-induced impairment of memory retrieval indeed results from inhibition of GABAergic interneurons through activation of their µRs, it is reasonable to expect that diminishing GABAergic synaptic transmission by blocking GABA_A_ receptors in the hippocampus will mimic the effects of stress on memory retrieval, whereas properly enhancing GABAergic transmission could counteract the effects of stress and attenuate the impairment. As shown in Fig. 5a, bilateral intra-hippocampal infusion of the GABA_A_ receptor antagonist bicuculline (6.0 ng/µl) 15 min before the probe test dramatically damaged memory retrieval in the unstressed mice to an extent similar to that observed in the stressed mice with saline infusion. It is well accepted that bicuculline non-selectively blocks GABA_A_ receptor-mediated phasic/tonic currents and L-655,708 selectively blocks the tonic inhibitory currents [33, 34]. Unlike the impairment observed with bicuculline, intraperitoneal administration of L-655,708 (0.7 mg/kg) or its vehicle (DMSO) 45 min before the probe test did not affect memory retrieval in the unstressed mice (Fig. 5a). It is thus reasonably concluded that depression of phasic inhibitory currents is the mechanism underlying the memory-retrieval impairment induced by EP stress.

We then studied the effect of muscimol, a GABA_A_ receptor agonist that can dose-dependently increase inhibitory currents [35, 36], on memory retrieval to further reinforce our theory. Because the over-activation of GABA_A_ receptors by muscimol at high concentrations may inhibit the normal function of cortical pyramidal neurons and disrupt learning and memory by inactivating or perturbing the internal theta rhythmicity of the network [37, 38], we set up our experiments with various concentrations of muscimol. Bilateral intra-hippocampal infusion of muscimol at both 1.5 ng/1.0 µl and 3.0 ng/1.0 µl 15 min before the probe test did not significantly affect memory retrieval in the unstressed mice, whereas each of the following three large dosages of 6.0 ng/1.0 µl, 12.0 ng/1.0 µl, and 24.0 ng/1.0 µl damaged memory retrieval (Fig. 5b). When a sub-effective dose of muscimol (1.5 ng/0.5 µl) was microinjected immediately after EP stress, memory retrieval remained unimpaired (Fig. 5c), suggesting that a small amount of muscimol is able to neutralize the stress-induced depression of GABAergic action by properly activating GABA_A_ receptors on pyramidal neurons. Muscimol putatively potentiates both phasic and tonic inhibitory currents in CA1 pyramidal neurons. To rule out the possibility that muscimol neutralizes memory-retrieval impairment through the potentiation of tonic current, we administered saline or L-655,708 (i.p.) 30 min prior to muscimol infusion. As shown in Fig. 5d, in probe tests carried out 45 min after EP stress, both saline + DMSO and saline + L-655,708 groups still exhibited the memory retrieval impairment, whereas the combined muscimol + L-655,708 group showed no observable impairment. Given that the treatments did not affect swim speed (Supplementary Figures 2c-f), these data strongly suggest that depression of inhibitory transmission onto CA1 pyramidal neurons is crucial in the memory-retrieval impairment induced by EP stress.

## Discussion

It has been well established that stress-induced impairments in learning and memory are associated with the direct effects of glucocorticoid hormones on CA1 glutamatergic synaptic plasticity [4–8, 39]. However, the present study revealed that in response to stress, EOPs are also responsible for the stress-induced impairment of memory retrieval through directly depressing CA1 GABAergic inhibitory interneurons. Furthermore, by using a combination of behavioural, biochemical, electrophysiological, and cell-type specific gene knockout techniques, the present paper provides several lines of coherent evidence, logically demonstrating that μRs expressed at GABAergic inhibitory neurons are activated by EOPs and subsequently depress the GABAergic synaptic transmission onto pyramidal neurons in the setting of stress-induced impairment of memory retrieval.

In response to stressful stimuli, activation of the autonomic nervous system (ANS) and the hypothalamic-pituitary-adrenal (HPA) axis will trigger the release of a series of stress-related hormones and neuromodulators. It is believed that in coping with stress, released EOPs can promote adaptation and benefit the organism by preventing the development of stress-related mental or physical disorders [10–12, 40]. In the hypothalamus, the corticotropin-releasing hormone (CRH) and the arginine vasopressin (AVP)-containing neurons in the paraventricular nuclei (PVN) are reciprocally connected with the proopiomelanocortin (POMC)-containing neurons in the arcuate nucleus. During stress, the CRH and AVP secreted from PVN neurons can activate POMC-containing neurons to release β-endorphin and other POMC-derived EOPs to a number of brain regions, including the hippocampus [11, 41]. It has been demonstrated that the hippocampal CA1 region contains an abundance of enkephalin-containing neurons and endorphinergic projections [42, 43]. Stress can also enhance the release of Met-enkephalin in the hippocampus [11, 44] to activate µRs or δRs [42]. Stress-induced release of EOPs can efficiently phosphorylate and internalize µRs, δRs or κRs with complicated mechanisms [45–48]. The present study shows that during acute stress, the enhanced hippocampal Met/Leu-enkephalin specifically activates µRs to reduce local inhibitory transmission (Fig. 3 and Fig. 4) and then impairs retrieval of spatial memory in mice (Fig. 1b and Fig. 1e). Using µR−/−, µR_GABA_−/−, µR_Glut_−/− and µR_Astro_−/− mice, we provide additional evidence demonstrating that it is the µRs expressed at GABAergic inhibitory neurons that mediate stress-induced impairment of memory retrieval by depressing local inhibitory neurotransmission. Although it remains elusive how EP stress increases met-enkephalin release in the hippocampus, the present paper provides the first detailed evidence for the essential role of EOPs in local CA1 circuitry in acute stress-induced memory impairments.

It has been reported that exogenous activation of µRs by either systemic morphine injection [49] or intra-hippocampal DAMGO microinjection (Fig. 1b) impaired memory retrieval in mice. However, the requirement of endogenous activation of µRs for normal spatial reference memory tasks was not observed using conventional µR knockout mice [50]. The present work also demonstrated that either pharmacological blockade of µRs (Fig. 1b) or knockout (whether non-selective or selective) of µR (Supplementary Figure 8) did not alter memory retrieval in the MWM task under unstressed conditions. In the case of the stressed condition, we observed that endogenous activation of µRs functionally damaged memory recall during acute stress. It has been shown that the involved µRs are localized exclusively at the terminals and somata of GABAergic inhibitory neurons and coupled with inhibitory G proteins. Activation of those µRs decreases GABA release presynaptically by a G protein-mediated inhibition process; [51] therefore, mIPSCs and sIPSCs recorded in pyramidal neurons innervated by those GABAergic neurons were reduced (Fig. 3). Meanwhile, binding with EOPs induces rapid phosphorylation of µRs on the intracellular C-terminal domains, which subsequently results in endocytosis of µRs in a β-arrestin-mediated manner within approximately 30 min [52]. Rapid phosphorylation of hippocampal µRs at the position of Ser375 followed by a reduction in the abundance of cell-surface µRs, which were putatively elicited by Met-enkephalin, was observed in the present study (Fig. 1c). The phosphorylation and internalization of µRs induced by stress blunt µR function and thus eliminate the DAMGO-induced inhibition of GABAergic transmission (Fig. 4b).

GABAergic interneurons are essential in balancing the excitation and inhibition signals in the brain. Disinhibition of hippocampal pyramidal neurons caused by depression of GABAergic neurons has been implicated in memory impairments [21] and psychiatric disorders [53]. Selective reduction in functional inhibitory synapses or GABA depletion in the dorsal hippocampus impairs spatial learning and memory [54]. Consistently, it was observed in the present work that blockade or over-activation of hippocampal GABA_A_ receptors impaired memory retrieval (Fig. 5a, b). In agreement with a previous study that showed that the GABA release probability is reduced in the hippocampus of stress-susceptible animals [55], our study revealed that in response to acute stress, the phasic inhibitory currents mediated by the intrasynaptic GABA_A_ receptors (containing α1, α2 and/or γ-subunits) were specifically suppressed. GABAergic inhibitory transmission in the CA1 field was depressed as a result of activation of µRs on GABAergic neurons (Fig. 3). We concluded that the reduction in phasic inhibitory currents is associated with stress-induced retrieval impairment because putatively upregulating the phasic component alone by co-administration of muscimol and L-655,708 prevented memory impairment after EP stress (Fig. 5d). GABAergic signalling can also be altered by chronic stress [27, 55–57] and thus interrupt the normal E/I balance in memory-related circuits in the brain. However, chronic stress simultaneously alters tonic inhibition and phasic inhibition [57–59]. How chronic stress extends its effects on GABAergic signalling from presynaptic to postsynaptic sites and from intrasynaptic to extrasynaptic GABA_A_ receptors is still unknown. A possible explanation is that acute stress preferentially alters GABA release by activating µR_GABA_, whereas the influence of chronic stress involves a long-lasting modulation of GABA_A_ receptors enacted by changing the distribution of their subunit compositions [60], altering their allosteric binding sites [60], and even disturbing astrocyte-mediated GABA uptake and degradation [61].

It is well known that memory retrieval depends both on individual neuronal firing behaviour and the synchronous oscillations of spatially distributed neurons [62]. A dynamic E/I balance is essential for maintaining neuronal firing behaviour and the oscillatory patterns of hippocampal neurons [63]. Breaking the E/I balance in either direction will damage memory retrieval by altering the firing threshold [64] or shifting the patterns of neural oscillation in the neuronal populations related to memory retrieval [65]. As observed in the present experiment, memory retrieval could be impaired by both blockade of the GABAergic receptors and activation of the receptors with high doses of agonists. During acute stress, non-selective disinhibition of hippocampal pyramidal neurons may change the firing rates of individual neurons and the oscillation patterns of neuronal populations, resulting in impairment of memory retrieval. In this case, muscimol application at a proper dosage might non-selectively neutralize the effects of stress on GABAergic inhibition and thereby rescue the impairment of memory retrieval.

It has been demonstrated that stress can impair memory retrieval by modulating glutamatergic synaptic plasticity in the hippocampus by inhibiting LTP induction or facilitating LTD expression [4–8]. A number of previous studies have observed that acute stress resets the thresholds for LTP and/or LTD, without an obvious effect on the basal excitatory synaptic efficacy in the CA1 region [7, 39, 66, 67]. Elevated corticosteroid hormones during stress facilitate LTD and suppress LTP, probably by regulating membrane trafficking of GluR2-containing AMPARs [68] and GluN2B-containing NMDARs [69], which can consequently lead to memory-retrieval impairments [4, 5, 7]. In addition to the corticosteroid-mediated modulation of synaptic plasticity, the present study clearly showed a new pathway in which acute stress depresses GABAergic inhibitory transmission to CA1 pyramidal neurons through endogenous activation of μR_GABA_, resulting in E/I imbalance in the hippocampus and thereby impairing memory retrieval. Intriguingly, our additional data [70] revealed that EP stress facilitates LTD induced by low-frequency stimulation (LFS, 900 pulses at 3 Hz) at the Schaffer collateral/commissural-CA1 synapses in mice and that this stress facilitated LFS-LTD depends on the activation of μR_GABA_ during stress (the facilitation of LTD can be abolished by naloxone administration before stress and is absent in mice lacking μR_GABA_). Facilitation of LTD suggests that activation of µR_GABA_ lowers the threshold of LTD induction (depolarization of pyramidal neurons removes shunting and hyperpolarizing effects on NMDAR activation) [71]. Thus, endogenous activation of µR_GABA_ by EP stress may not only disturb E/I balance but also modulate synaptic plasticity, both of which are associated with hippocampus-based memory retrieval [72, 73]. These results suggest that corticosterone-mediated modulation of synaptic plasticity is not the sole pathway of memory-retrieval impairments induced by stressful events [9, 74, 75] (Fig. 5e). Blocking this µR-mediated depression of local inhibitory transmission reversed the memory impairment but had no effects on the elevation of corticosterone induced by stress (Fig. 1g), implying that in response to acute stress, this µR-mediated depression of local inhibitory transmission is sufficient to cause memory-retrieval impairment. However, the possible effects of stress hormones on the secretion of EOPs and activation of the µR-mediated depression of inhibitory transmission in the hippocampus remain to be addressed in future.

## Supplementary information

Supplementary Materials
