# Supplementary material for: Hippocampal µ-opioid receptors on GABAergic neurons mediate stress-induced impairment of memory retrieval
Source: Mol Psychiatry. 2019 May 29;25(5):977–92. doi: 10.1038/s41380-019-0435-z (PMC7192851; doi:10.1038/s41380-019-0435-z)
Supplement: Supplementary file 1 — Supplementary Materials [file 41380_2019_435_MOESM1_ESM.docx]

**Hippocampal µ-opioid receptors on GABAergic neurons mediate stress-induced impairment of memory retrieval**

**Supplementary materials**

**
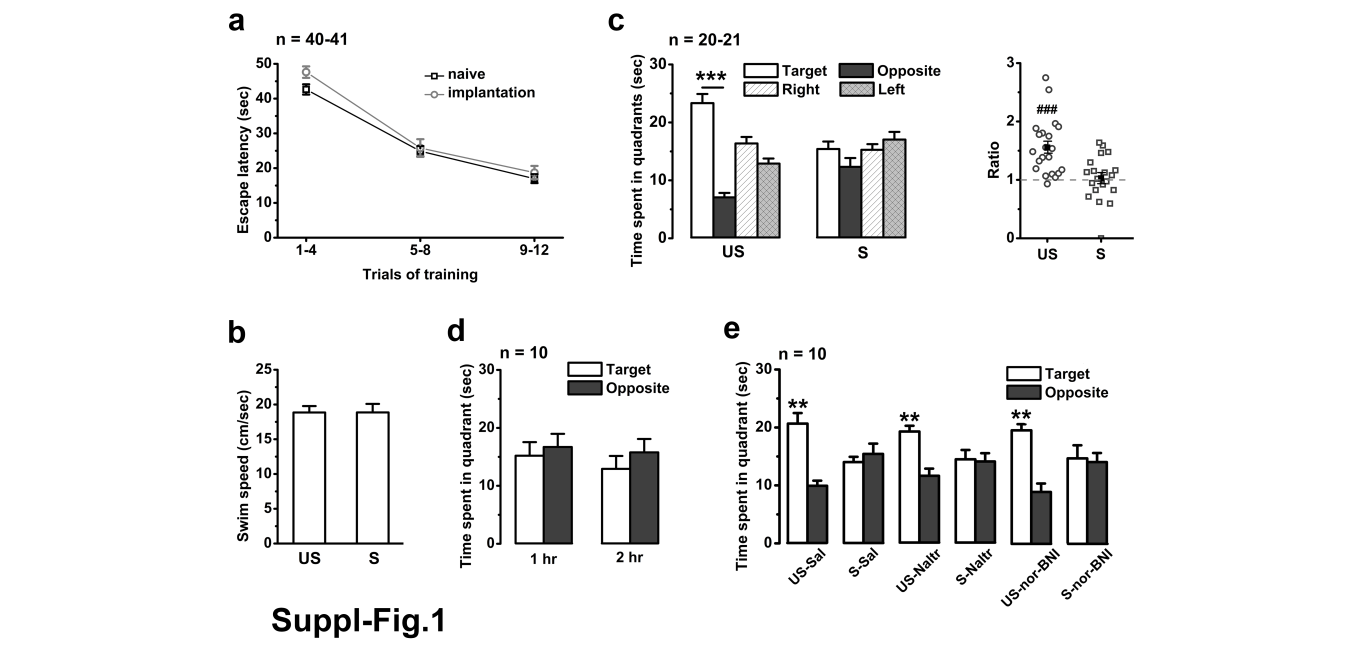
**

**Supplementary Figure 1** Acute EP stress before the MWM probe test impairs the retrieval of spatial reference memory in mice. (a) The escape latency of naïve and guide cannulas implanted mice during 12 trials of MWM training. Mice of naïve and implantation showed similar escape latencies, which decreased gradually with the increased training trials in both groups. Group *F*_1,79_ = 1.66, *p* = 0.201; trial *F*_2,158_ = 229.82, *p* = 0.000; group×trial *F*_2,158_ = 1.34, *p* = 0.266; RM ANOVA. (b) Stress does not affect the average swimming speed during the probe test. *p* = 0.996, unpaired Student’s *t*-test. (c) The effects of stress on the memory retrieval of trained naïve mice immediately prior to the probe test. Left, time spent in the four quadrants during the test trial. Target versus opposite within-group, ****p* < 0.001, paired Student’s *t*-test. Right, the target time ratio. ###p < 0.001, unpaired Student’s *t*-test. (d) The probe tests were carried out 1 hour and 2 hours after stress. *p* = 0.748 for 1 hour and *p* = 0.505 for 2 hours, paired Student’s *t*-test. (e) Dose enhancement of naltrindole (4.52 µg/µl) or nor-binaltorphimine (7.70 µg/µl) does not affect stress-induced impairment of memory retrieval. ***p* < 0.01, paired Student’s *t*-test. US, unstressed; S, stressed; Sal, saline; Naltr, naltrindole; nor-BNI, nor-binaltorphimine.


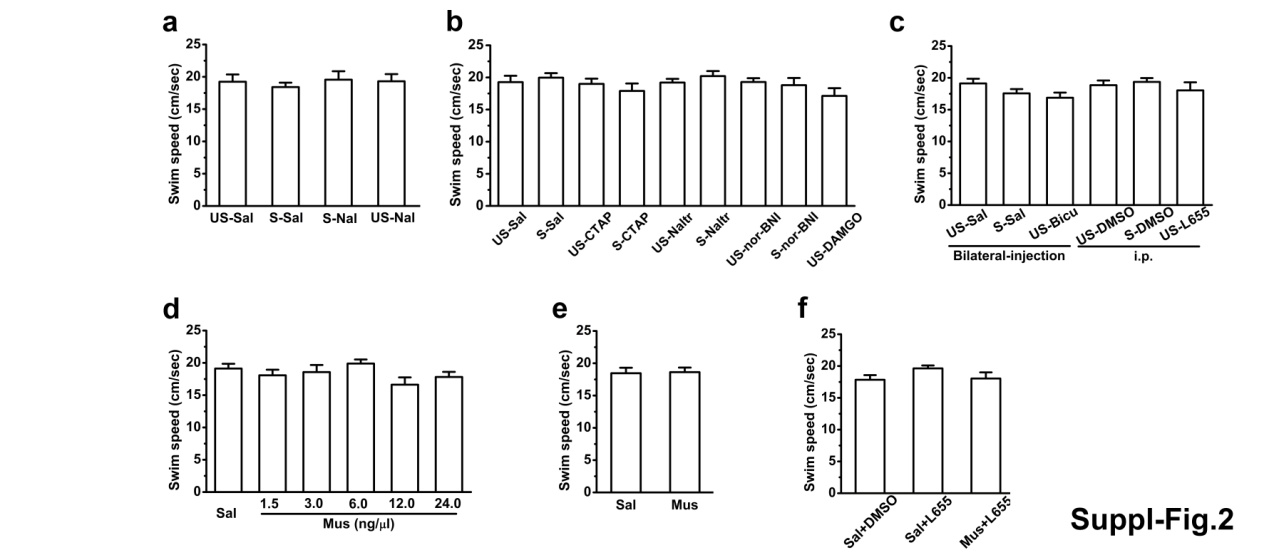


**Supplementary Figure 2** The average swim speed during the probe test of MWM under various conditions. One-way ANOVA or Student’s *t*-test; n = 10 for each group. (a) Systemic injection of naloxone or saline in unstressed and stressed mice. *F*_3,36_ = 0.01, *p* = 0.999. (b) Bilateral intra-hippocampal infusion with CTAP, naltrindole, nor-binaltorphimine, DAMGO, or saline on the unstressed and stressed mice. *F*_8,81_ = 1.00, *p* = 0.443. (c) Bilateral intra-hippocampal infusion with bicuculine or intraperitoneal administration of L-655,708 on the unstressed mice. *F*_5,54_ = 1.36, *p* = 0.253. (d) Bilateral intra-hippocampal infusion with different doses of muscimol on the unstressed mice. *F*_5,34_ = 1.63, *p* = 0.167. (e) Bilateral intra-hippocampal infusion with muscimol (1.5 ng/1.0 µl) on the stressed mice. *p* = 0.883. (f) The combined muscimol + L-655,708 injection on the stressed mice. *F*_2,27_ = 1.74, *p* = 0.195. US, unstressed; S, stressed; Sal, saline; Nal, naloxone; Naltr, naltrindole; nor-BNI, nor-binaltorphimine; Bicu, bicuculine; L655, L-655,708; Mus, muscimol.

**
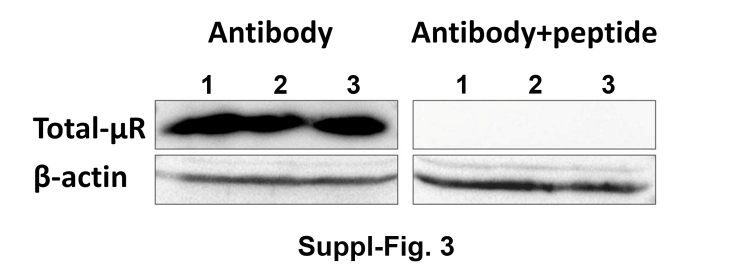
**

**Supplementary Figure 3** Verification of the µR-antibody specificity used in experiments by Western blots. The immunoreactivity of hippocampal tissue extracts is no longer detectable when the antibody is mixed with peptides (n = 3 for each group).

**
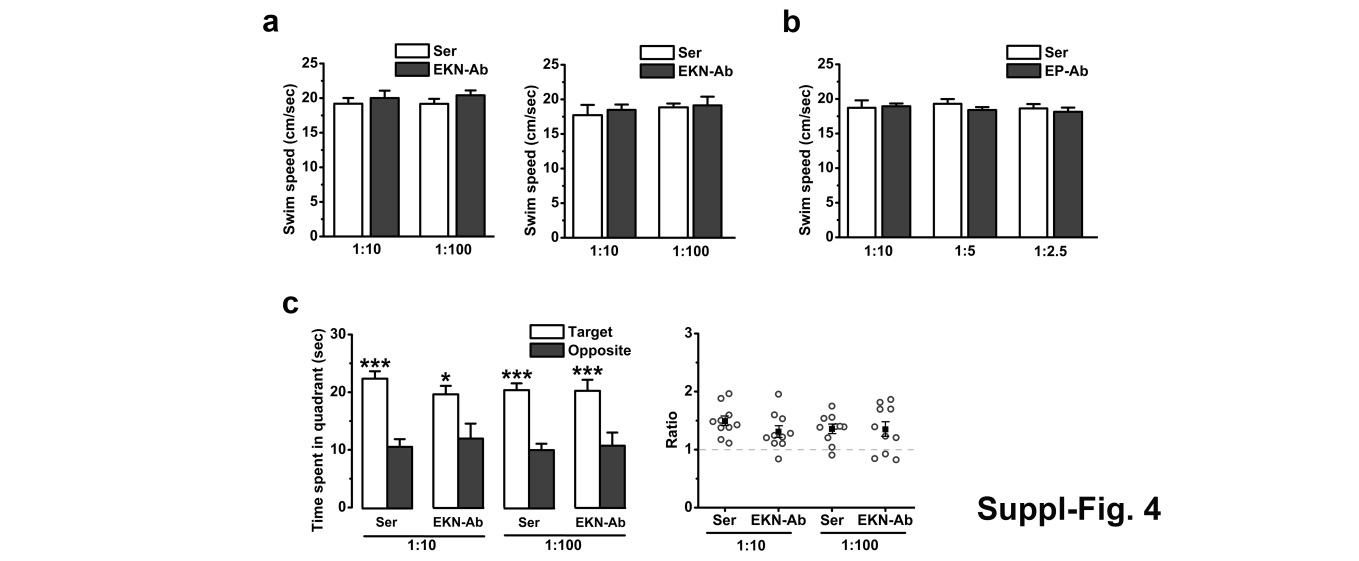
**

**Supplementary Figure 4** The effects of hippocampal injection of β-endorphin/enkephalin antiserum on memory retrieval and locomotor activity. One-way ANOVA or paired Student’s *t*-test; n = 10 for each group. (a) The average swim speed of the unstressed (left) and stressed (right) groups in the test trial after enkephalin antiserum or control serum microinjection. Unstressed *F*_3,36_ = 0.54, *p* = 0.656; stressed *F*_3,36_ = 0.32, *p* = 0.813. (b) Swimming speed of the stressed mice in the test trial after β-endorphin antiserum or control serum microinjection. *F*_5,54_ = 0.36, *p* = 0.873. (c) Microinjection of enkephalin antiserum 110 minutes before the probe test does not alter memory retrieval in the unstressed mice. Target versus opposite within-group (left); the ratio of time spent in target quadrant (right) *F*_3,36_ = 0.62, *p* = 0.604. One symbol, *p* < 0.05; three symbols, *p* < 0.001. EKN-Ab, enkephalin antiserum; EP-Ab, β-endorphin antiserum; Ser, control serum.

**
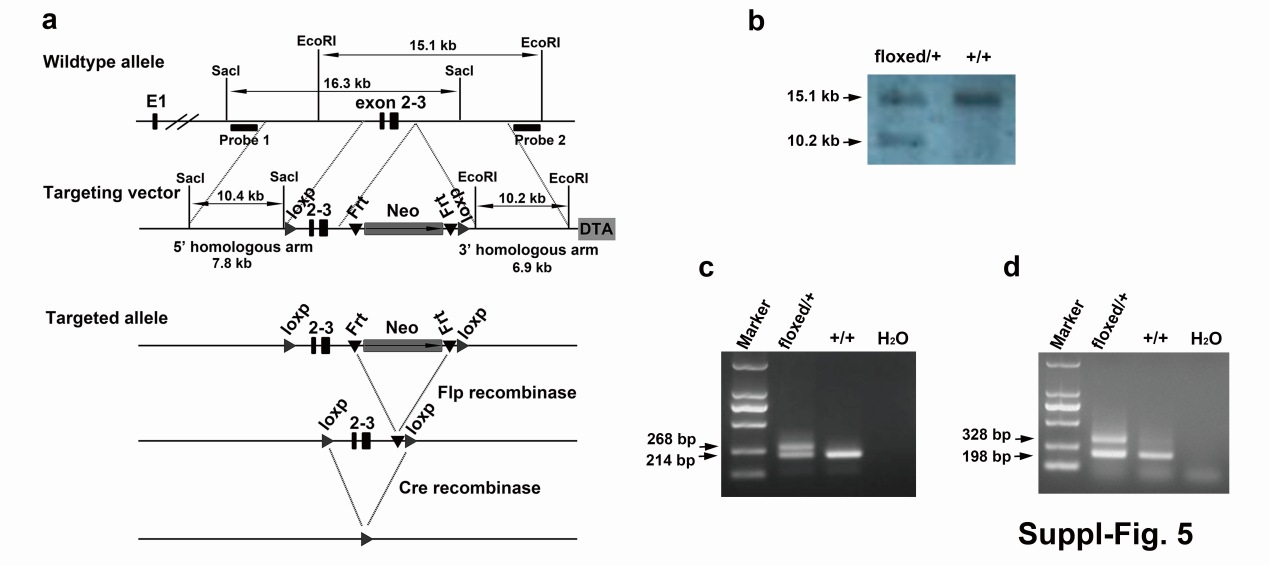
**

**Supplementary Figure 5** Generation of mice with floxed *Oprm1* allele. (a) Schematic representatives of the wild-type *Oprm1* allele (exons 2-3) in mice, targeting vector and floxed *Oprm1* allele after being crossed with Flp-deletion mice. The 7.8 kb fragment upstream of exons 2-3 and the 6.9 kb fragment downstream are used as the 5′ and 3′ homologous regions, respectively. (b) Southern blot analysis shows that once homologous recombination is completed, the EcoRI-digested fragment would turn from 15.1 kb to 10.2 kb as detected with a probe. (c) The offspring were genotyped by PCR using primers of *Oprm1*-LoxP-F and *Oprm1*-LoxP-R. The 214-bp and 268-bp bands indicate the wild-type allele (+) and the floxed allele, respectively. (d) The offspring are genotyped by PCR using primers of *Oprm1*-Frt-F and *Oprm1*-Frt-R. The 198-bp and 328-bp bands indicate the wild-type allele and the floxed allele, respectively.

**
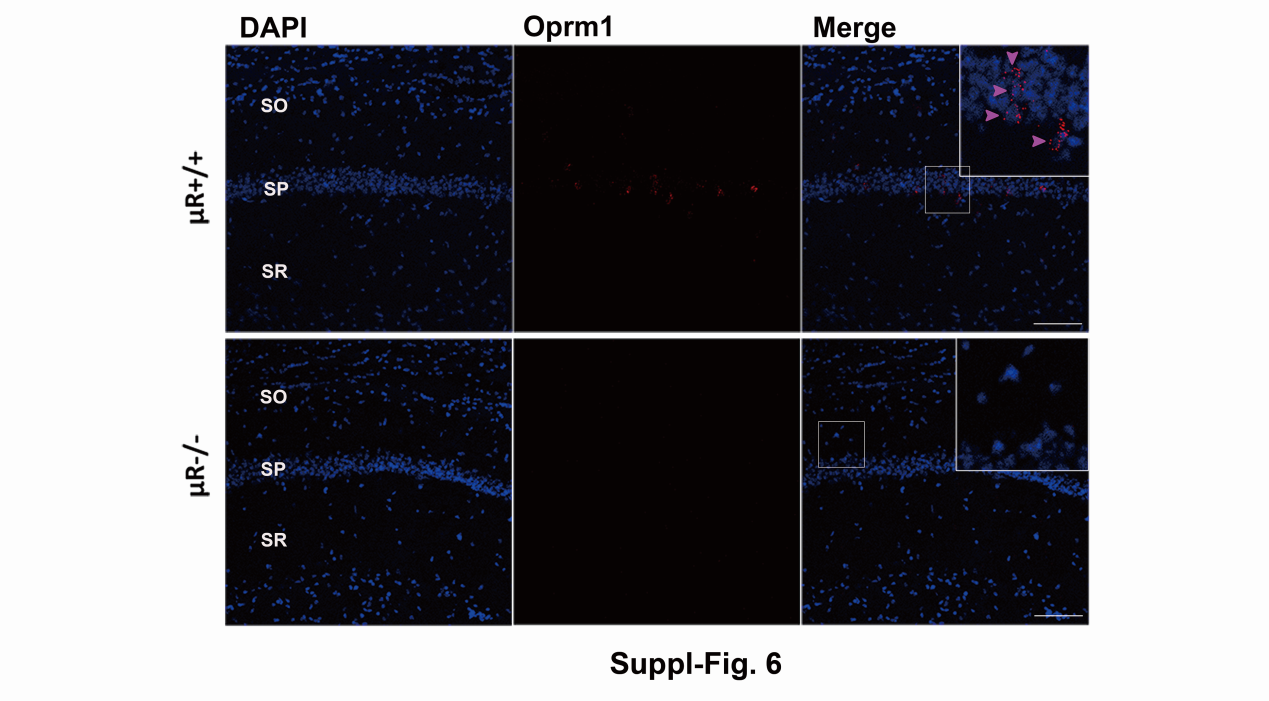
**

**Supplementary Figure 6** *In situ* hybridization in the hippocampal CA1 area to confirm µR deletion from µR-/- mice with a probe of µR mRNA. The nucleus is stained in blue (DAPI), and *Oprm1* mRNA is shown in red. Insets are higher-magniﬁcation images of the fields indicated by white boxes. Purple arrowheads represent µR mRNA-positive cells in µR+/+ mice, which cannot be observed in µR-/- mice. SO, stratum oriens; SP, stratum pyramidale; SR, stratum radiatum. Scale bar, 100 μm.

**
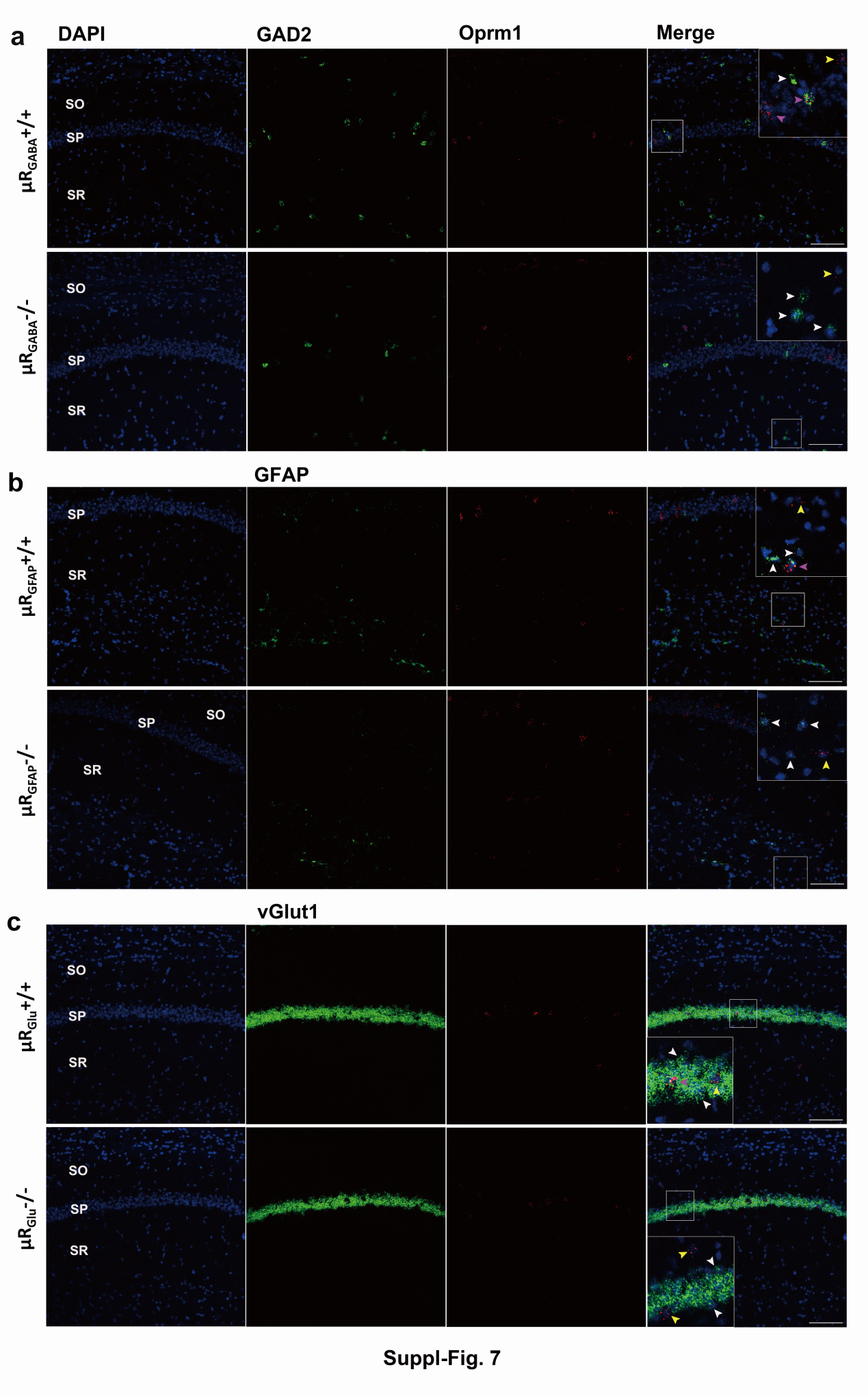
**

**Supplementary Figure 7** *In situ* hybridization in hippocampal CA1 area to confirm µR selective deletion from µR_GABA_-/- (a), µR_GFAP_-/- (b), µR_Glu_-/- (c) mice with probes of µR mRNA (red), GAD2/ GFAP/vGlut1 mRNA (green), and DAPI nucleus staining (blue), respectively. Insets are higher-magniﬁcation images of the fields indicated by white boxes. Purple arrowheads represent double-labelled cells with µR mRNA and GAD2, GFAP, or vGlut1 mRNA, respectively; yellow arrowheads represent µR mRNA localization in GAD2, GFAP, or vGlut1 negative cells; white arrowheads represent GAD2, GFAP, or vGlut1 positive cells without µR mRNA. µR mRNA is enriched in GAD2-positive cells, with minor expression in GFAP- or vGlut1-positive cells. Please note double-labelled cells in control mice are absent in related µR deleted mice. SO, stratum oriens; SP, stratum pyramidale; SR, stratum radiatum. Scale bar, 100 μm.

**
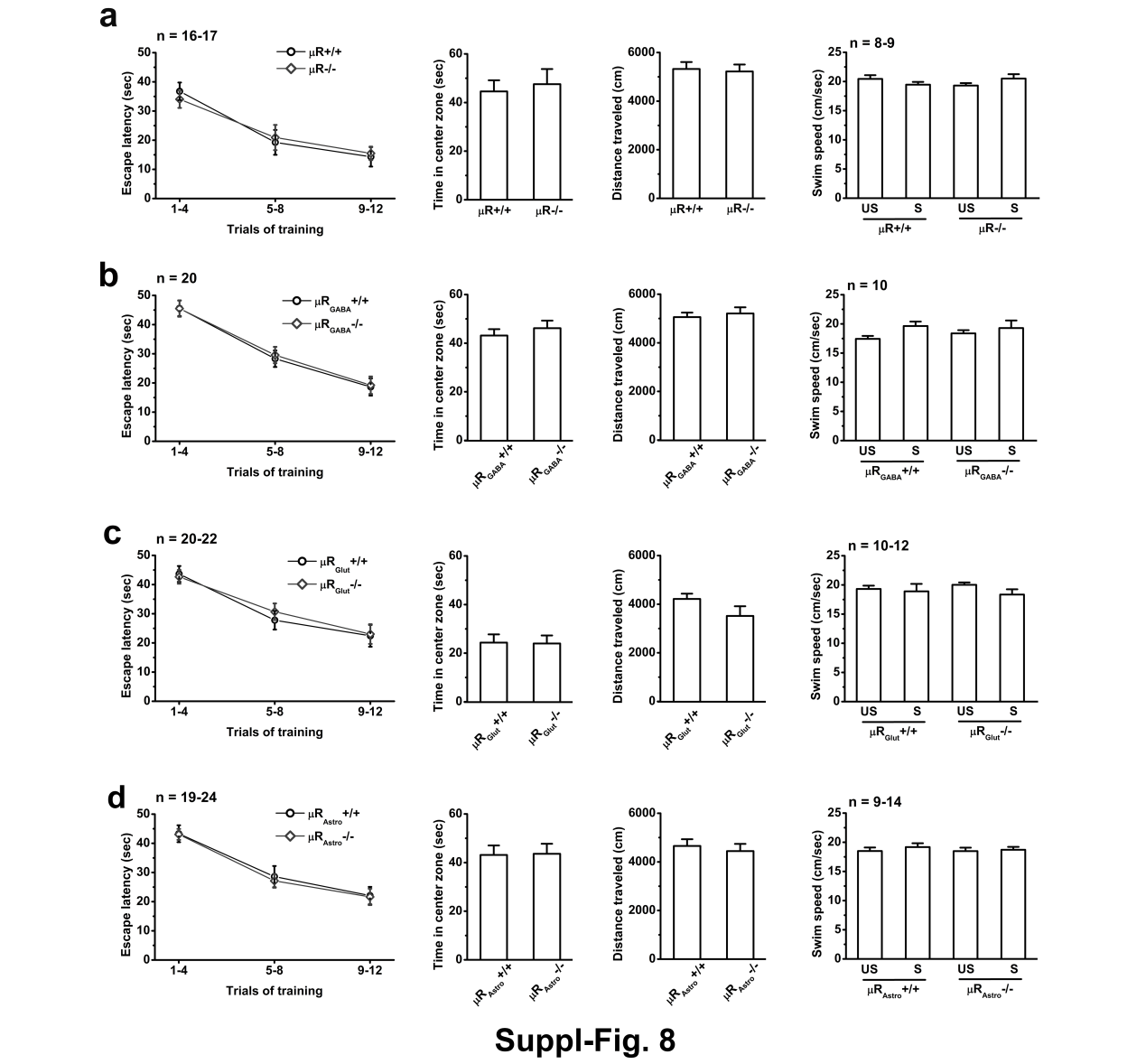
**

**Supplementary Figure 8** Non**-**selective or selective deletion of µR does not significantly affect the learning capability or emotional and motor activities of mice. Experiments involving animals of each genotype are performed 2 weeks after 7 daily injections of tamoxifen. (a) Escape latency during MWM training (genotype *F*_1,31_ = 0.00, *p* = 0.993; trial *F*_2,62_ = 67.94, *p* = 0.000; genotype×trial *F*_2,62_ = 0.61, *p* = 0.520), time spent in the centre zone (*p* = 0.202) and total distance travelled (*p* = 0.804) during the OF test, and the average swim speed in the MWM probe test (*F*_3,36_ = 1.39, *p* = 0.263) for the µR conditional knockout line. (b) Escape latency during MWM training (genotype *F*_1,38_ = 0.04, *p* = 0.844; trial *F*_2,76_ = 67.94, *p* = 0.000; genotype×trial *F*_2,76_ = 0.04, *p* = 0.959), time spent in the centre zone (*p* = 0.464) and total distance travelled (*p* = 0.636) during the OF test, and the average swimming speed in the MWM probe test (*F*_3,36_ = 1.39, *p* = 0.263) for the µR_GABA_ line. (c) Escape latency (genotype *F*_1,40_ = 0.06, *p* = 0.810; trial *F*_2,80_ = 43.19, *p* = 0.000; genotype×trial *F*_2,80_ = 0.38, *p* = 0.685), time spent in the centre zone (*p* = 0.936) and total distance travelled (*p* = 0.631), and the average swim speed in the MWM probe test (*F*_3,38_ = 0.67, *p* = 0.573) for the µR_Glut_ line. (d) Escape latency (genotype *F*_1,41_ = 0.06, *p* = 0.805; trial *F*_2,82_ = 54.08, *p* = 0.000; genotype×trial *F*_2,82_ = 0.04, *p* = 0.961), time spent in the centre zone (*p* = 0.936) and total distance travelled (*p* = 0.631), and the average swim speed in the MWM probe test (*F*_3,39_ = 0.26, *p* = 0.853) for the µR_Astro_ line. Escape latency with repeated-measures ANOVA, time spent in the centre zone and total travel distance analysed with an unpaired Student’s *t*-test; average swimming speed analysed with one-way ANOVA. US, unstressed; S, stressed.
